# Immunohistochemical Analysis of IL-19 and IL-24 Expression in Inflammatory Bowel Disease (IBD) Patients: Results From a Single Center Retrospective Study

**DOI:** 10.7759/cureus.64441

**Published:** 2024-07-12

**Authors:** Alexandros Toskas, Stephanos Milias, Georgios Delis, Soultana Meditskou, Antonia Sioga, Sofia Karachrysafi, Theodora Papamitsou

**Affiliations:** 1 Histology, Aristotle University of Thessaloniki, Thessaloniki, GRC; 2 Histopathology, Private Histopathology Laboratory, Thessaloniki, GRC; 3 Veterinary Medicine, Aristotle University of Thessaloniki, Thessaloniki, GRC; 4 Histology and Embryology, Aristotle University of Thessaloniki, Thessaloniki, GRC; 5 Medicine, "George Papanikolaou" General Hospital of Thessaloniki, Thessaloniki, GRC; 6 Histology and Embryology, Aristotele University of Thessaloniki, Thessaloniki, GRC

**Keywords:** biologics, immunochemistry, uc, crohns, ibd, il-19, il-24

## Abstract

Background

IL-19 and IL-24 induce proinflammatory cytokine production through the Janus kinase-signal transducer and activator of transcription (JAK-STAT) pathway. The primary objective of this study was to investigate any changes in IL-19 and IL-24 expression between inflammatory bowel disease (IBD) patients and healthy controls, as well as before and after the initiation of biologics. The secondary objective was to investigate any relation between their expression and disease phenotype and activity.

Methods

IL-19 and IL-24 expression was measured in intestinal tissue samples from 121 patients with moderate to severe IBD versus healthy controls using immunohistochemistry. Their expression was then measured 12 months after treatment on the patient group treated with biologics. The disease activity was measured before and after treatment using the Harvey Bradshaw Index (HBI) for Crohn's disease (CD) patients and the Mayo Score (MS) for ulcerative colitis (UC) patients. Data were analyzed using SPSS (IBM Inc., Armonk, New York).

Results

IL-19 expression was raised in the IBD group versus healthy controls. In the CD group, the IL-19 expression was related with the disease activity score post-biologic treatment. IL-24 was also highly expressed in patients with active UC and CD and was increased post-treatment. Its expression in UC was statistically related with the MS.

Conclusions

IL-24 and IL-19 are key factors in IBD-related intestinal inflammation and this is one of the few human studies to suggest that. An immunosuppressive role of IL-24 was demonstrated in the UC group. A future use as biomarkers of disease activity and response to treatment might be feasible.

## Introduction

Novel anti-interleukin drugs have been recently approved for the treatment of inflammatory bowel disease (IBD), highlighting the importance of studying the role of certain interleukins in disease pathophysiology. The immune response against gut flora is most likely one of the main trigger factors for Crohn's disease (CD) and ulcerative colitis (UC). T helper (Th) 1 immune cell reaction, involving interferon-gamma (IFN)-γ and interleukin-12 (IL-12), is the main pathophysiologic feature of CD and Th-2 response, characterized by the production of IL-5 and IL-13, for UC [1].

IL-19 and IL-24 are both part of the IL-10 cytokine group and they share the same receptor (IL-20 receptor b-subunit-IL-20 RB) to signal through [2]. IL-10 is an anti-inflammatory cytokine, with animal studies suggesting that it can maintain tissue homeostasis and ameliorate tissue inflammation. Previous studies suggested that it can also promote the differentiation and activity of T-regulatory (T-reg) cells [3].

IL-24 signals through the Janus kinase-signal transducer and activator of transcription (JAK-STAT) pathways and the suppressor of cytokine signaling-3 (SOCS-3) pathway. It regulates the expression of clusters of differentiation 4+ cells (CD4+), CD8+, Natural Killer cells (NK), B-cells, and macrophages [2,4,5]. IL-24 can also increase mucin expression, demonstrating a protective effect against mucosal inflammation. However, its role in human inflammatory diseases is not yet clear [2]. In patients with psoriasis and rheumatoid arthritis, previous studies have suggested that it increases pro-inflammatory cytokines, mainly IL-6 and tumor necrosis factor-alpha (TNF-a) [5]. However, conflicting studies have shown a rather tolerogenic effect in IBD and liver inflammation [2].In a few human studies involving patients with active IBD, IL-24 was found to be expressed in the inflamed intestinal mucosa. It was also suggested that it helped to protect and maintain the integrity of the mucosal epithelium [5-8].

IL-19 is an activator of IL-10 transcription, and therefore, there is a hypothesis that might ameliorate active colitis; however, there is no sufficient data to support this. Several cell types produce IL-19, especially keratinocytes, epithelial cells, monocytes, and B cells [9]. When it binds to its' heterodimeric receptor (IL-20Ra/IL-20Rb), it triggers the STAT pathway, notably STAT1 and STAT3. The role of IL-19 has been mainly investigated in psoriasis, suggesting that it likely contributes to colonic inflammation [10]. Data from a few animal studies showed that IL-19 might control intestinal inflammation and induce mucosal healing in experimental colitis models [7]. Other studies showed that certain polymorphisms of IL-19 had a protective effect in UC patients [9, 11, 12]. In other studies, IL-19 expression was raised in intestinal biopsies of patients with active CD in comparison with UC patients or healthy controls [9, 11, 13].

In this study, we aim to investigate any difference in the expression of IL-19 and IL-24 between IBD patients and healthy controls, as well as any changes before and after biologic treatment, using immunohistochemistry. We also aim to identify a possible relation between their expression, the disease phenotype, and activity.

## Materials and methods

Data collection

To perform this study, we used the same cohort of patients as previously mentioned by Toskas et al. [14] and the same methodology for data collection. A personal history of 121 patients with IBD was taken from the archives of the Gastroenterology Department of 424 General Military Hospital of Thessaloniki between 1999 and 2020. Demographic data and information regarding disease extent and severity were recorded. The treatment options offered and the endoscopic reports before and 12 months after treatment with biologics were retrieved. Intestinal biopsies before and one year after treatment with biologics were retrieved from the histopathology lab for further analysis. Signed consent was received from all the patients involved, and the study was approved by the Bioethics Committee of Aristotle University of Thessaloniki [14].

Study groups

The initial sample was divided into two groups: A) CD patients and B) UC patients. Subsequently, each of these two groups was divided into three subgroups based on the biologic treatment the patients had: 1) anti-tumour necrosis factor alpha (anti-TNFa) drug, adalimumab (ADA) and infliximab (IFX), 2) ustekinumab (USK), 3) vedolizumab (VDZ). The activity of the disease was measured before and one year after treatment using the Harvey-Bradshaw Index (HBI) for CD and the Mayo Score (MS) for UC. The activity of the disease in the group that received conventional treatment (aminosalicylates, steroids, or thiopurines) was measured before treatment. Intestinal biopsies from twenty patients without an IBD history were used as controls. These 20 patients were investigated for chronic diarrhea and had a normal colonoscopy with normal screening biopsies, without features of colonic inflammation [14].

Immunohistochemistry

For the purposes of immunohistochemistry, the IL-19 rabbit polyclonal antibody (89132, NOVUS Biologicals Ltd, Oxon, UK) and the IL-24 rabbit polyclonal antibody (ab115207 ABCAM Ltd, Cambridge, UK) were used, after dilution with an antibody buffer solution. The IL-19 was diluted in 1:100 concentration and the IL-24 in 1:50. The same immunohistochemical (IHC) staining process was used for each of the two antibodies tested, as described below [14].

One paraffin block for each patient was retrieved from the archives and studied. Two sections of each block (4 μm in thickness) were cut and fixed in unstained, positively charged histology glass slides. A positive control from lymph node tissue and a negative control from pancreatic tissue were also cut and fixed in similar slides [14]. These were deparaffinized in the incubation chamber (Thermo^TM^ Scientific, Massachusetts, USA) at 60.50 ^o^C for 45 minutes. The immunohistochemistry protocol was completed with a BOND^TM^ polymer refined detection kit using the IHC protocol F [14]. Staining of the slides was performed with an automatic immunostainer Bond-MAX^TM^ (Leica^TM^ biosystems) using the following protocol: 1) heat-induced epitope retrieval (HIER) for 30 minutes (for both antibodies); 2) blocking of peroxide for five minutes; 3) marker incubation for 15 minutes; 4) post-primary for eight minutes; 5) polymer for eight minutes; 6) mixed 3,3'-diaminobenzidine (DAB) for 10 minutes; and 7) hematoxylin for five minutes [14]. At the end of each cycle, the slides were dehydrated in alcohol solution (75^o^, 95^o^, 100^o)^, cleared with xylene, and then covered with slide covers with mounting medium [14]. An optical Nikon^TM ^microscope with an attached Euromex^TM^ scientific camera was used for microscopic evaluation [14]. The intensity of staining was evaluated using a semi-quantitative method (combination of the intensity of staining and the percentage of positive cells), as negative (-), weak (+), moderate (++), or strong (+++), by an experienced gastrointestinal (GI) histopathologist with a special interest in IBD [14].

Statistical analysis

Data were analyzed using descriptive and regression statistics. The age, duration, activity, and treatment choices of IBD patients were analyzed using descriptive statistics. The IL-19 and IL-24 expression between controls and IBD patients prior to treatment were analyzed using ANOVA. The Kruskal-Wallis non-parametric test was used to compare the intensity of staining between subgroups that were treated with biologics, before and after treatment. SPSS version 29 (IBM Inc., Armonk, New York) was used for statistics and table creation [14].

## Results

Seventy-two patients with CD and 49 patients with UC were studied. The majority had a disease duration of ≥3 years (Appendix 1). The biologic group consisted of 71 patients (19 with UC, 52 with CD). Of the UC patients, 83.7% had moderately and severe UC on presentation. Of Crohn's patients, 79.2% had moderately and severely active disease (Appendices 2,3) [14].

IL-19 expression

IL-19 Expression in IBD Patients

**Figure 1:**
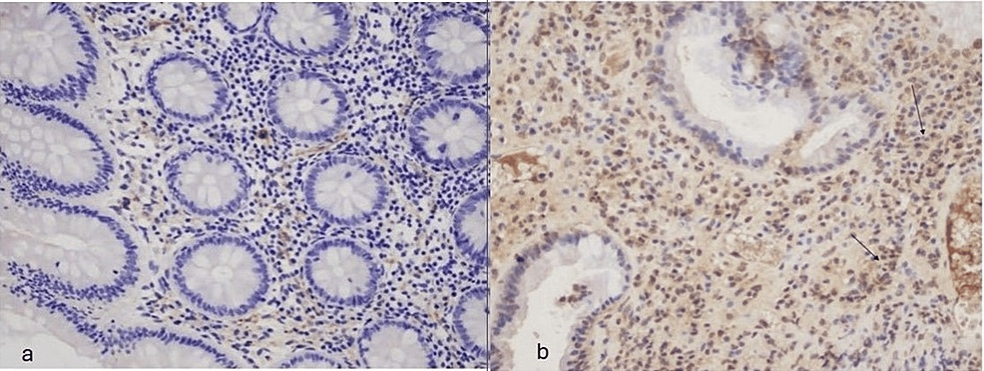
IL-19 expression in a non-IBD control (a) and in a patient with severe Crohn's colitis (b) a) Weak (+) expression of IL-19 (x200); b) Strong (+++) expression of IL-19 (x200) IL-19 - interleukin-19; IBD - inflammatory bowel disease

Pre-treatment IL-19 was significantly increased in the IBD group (N=121) versus non-IBD controls (N=20; p<0.05; Figure 1). 

In IBD group treated with biologics (N=71), there was a positive statistically significant correlation between the pre-treatment IL-19 expression and the score reduction (Δscore; p<0.01). A significant correlation was also found between the difference in IL-19 expression (ΔIL19) in the anti-TNFa-treated subgroup (p<0.05; N=59) and score reduction (Δscore; Appendices 4,5).

IL-19 Expression in UC Patients

**Figure 2:**
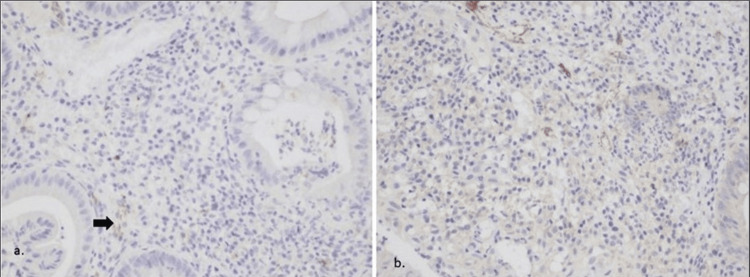
Colonic tissues from a UC patient treated with infliximab a) Prior to treatment, weak (+) expression of IL-19 (x 200); b) Post-treatment, negative (-) expression of IL-19 (x200) IL-19 - interleukin-19; UC - ulcerative colitis

No difference was found in pre-treatment IL-19 expression among different classes of Montreal extent and severity or activity of the disease on presentation, based on Mayo score (MS). Also, no difference was found between the expression of IL-19 in UC patients (N=49) and controls. No correlation was found between UC extent or severity and IL-19 expression. In the UC patient group treated with biologics (N=19), there was no relation between IL-19 expression before or after treatment and MS post-treatment. Data from this group were divided according to the biologic given, and those indices were checked again using the Kruskal-Wallis non-parametric test. No difference was found between MS reduction and IL-19 expression before and after treatment in these subgroups equally (Figure 2).

IL-19 Expression in CD Patients

The IL-19 expression was significantly higher in the CD group of patients (N=72) in comparison with non-IBD controls (P<0.01). IL-19 expression was not related with the disease phenotype as per Montreal L or B classification.

**Figure 3:**
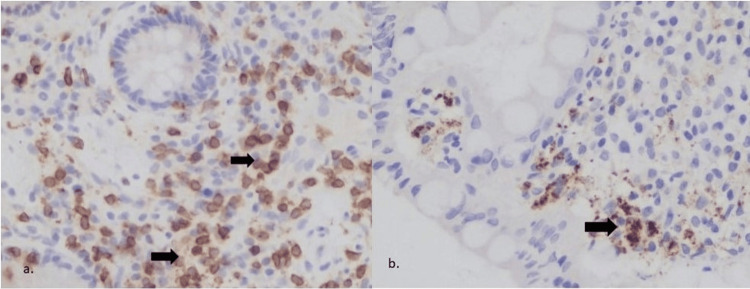
Intestinal biopsies from a CD patient pre- (a) and post- (b) infliximab treatment (a) Strong (+++) intensity of staining for IL-19,  x200; b) Moderate (++) staining for IL-19, 12 months post treatment with infliximab, x200 IL-19 - interleukin-19; CD - Crohn's disease

In the CD group of patients treated with biologics (N=52), a positive statistically significant correlation was found between the pre-treatment IL-19 expression and the HBI score reduction post-treatment (p <0.01, Spearman's correlation=0.362). A reverse statistically significant correlation was also found between the difference of IL-19 expression (ΔIL-19) in the anti-TNFa (N=44) and vedolizumab treated (N=2) subgroups and the HBI index reduction (p<0.05; Figure 3).

IL-24 expression

IL-24 Expression in IBD Patients

The pre-treatment IL-24 expression was significantly raised in the IBD patient group (N=121) versus healthy controls (p<0.05). IL-24 expression was significantly increased post-treatment in patients treated with biologics (N=71) and, more specifically, with anti-TNFa agents (N=59), but not in the other subgroups (p<0.01; Appendices 4, 5).

IL-24 Expression in UC Patients

The pre-treatment IL-24 expression in UC patients (N=49) was not increased compared with controls, and there was no relation between different classes of disease extents or severity.

**Figure 4:**
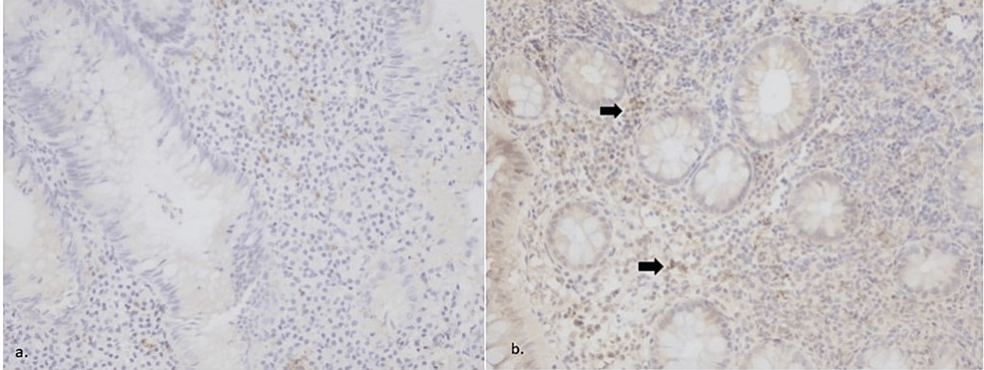
Colonic tissues from UC patient pre- (a) and post- (b) treatment with infliximab a) Negative (-) tissue staining for IL-24  pre-treatment; b) Weak (+) intensity of staining post-treatment, x200 IL-24 - interleukin-24; UC - ulcerative colitis

In the UC group (N=49), IL-24 expression post-treatment was statistically increased in patients with a reduction in their MS (p<0.05). This is indicative of a likely anti-inflammatory effect of IL-24 on the inflamed colonic mucosa (Figure 4).

The pre-treatment IL19 expression was also significantly correlated with the IL-24 expression before treatment (p<0.001; r=0.472)

IL-24 Expression in CD Patients

No difference was found in IL-24 expression between CD patients (N=72) and healthy controls. IL-24 expression was not related with disease activity or extent.

**Figure 5:**
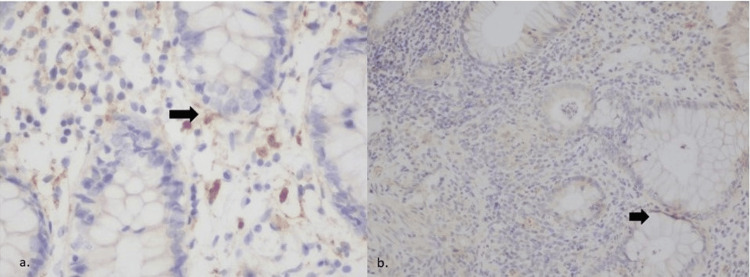
Intestinal tissue samples from CD before (a) and after (b) treatment with adalimumab a) Weak (+) staining for IL-24 before adalimumab treatment, x400. b) Weak (+) tissue staining for IL-24 post adalimumab treatment, x200 IL-24 - interleukin-24; CD - Crohn's disease

On the anti-TNFa treated subgroup (N=44) there was a significant relation between pre- and post-treatment IL-24 expression (p<0.01; Figure 5).

## Discussion

To our knowledge, this is the first retrospective single-center study regarding IL-19 and IL-24 expression in human intestinal tissue before and after treatment with biologics. So far, only a few studies investigated the expression of IL-19 and IL-24 in the colonic mucosa of IBD patients, and their role remains unclear [9, 11].

IL-24 was demonstrated to act differently in different types of autoimmune diseases, and dysregulation of its expression was related with autoimmunity. However, in some conditions, it showed a likely anti-inflammatory effect, like in IBD and liver inflammation [2]. Previous studies have shown that its levels are increased in the inflamed colonic mucosa. Andoh et al. detected increased IL-24 expression from the subepithelial myofibroblasts of the inflamed colonic mucosa of IBD patients, which was not found in patients with ischaemic or infectious colitis [5]. Similarly, Camarillo et al., in their cross-sectional study, showed that IL-24 was highly expressed in the colonic mucosa of both UC and CD patients. This was confirmed by using quantitative real-time polymerase chain reaction (RT-PCR) to measure the IL-24 mRNA and immunohistochemistry as well. In this study, the expression of IL-24 was found to be significant in CD patients in comparison with UC and healthy controls [7]. Its receptor, IL-20R2, was also upregulated in active UC. In an older study, Fonseca-Camarillo et al. demonstrated a high IL-20R2 gene expression in the inflamed colonic mucosa of a small group of 40 UC patients using RT-PCR [15]. Our study agrees with those results, showing an increased IL-24 expression in IBD patients with active inflammation versus healthy controls (p<0.05).

Further to that, the IL-24 expression was found to be statistically increased one year post-biologic treatment in the IBD group, especially in the anti-TNFa treated subgroup (P<0.01). This indicates that IL-24 possibly ameliorates colonic inflammation. Andoh et al. study support our results, suggesting that the colonic epithelial cells are a target for IL-24, but also that in human colonic epithelial cell lines, IL-24 activates the JAK-STAT3/SOCS-3 immunosuppressive pathway. It also stimulates the mRNA increase of Mucin (MUC) 1,3,4 in these cells, which enhances the intestinal barrier function. Further to that, there was no mRNA increase of proinflammatory cytokines like IL-6 and TNF-α [5].

Our data are also in agreement with previous animal experimental colitis studies that showed similar results. Onody et al. demonstrated that IL-24 expression was increased in both colonic and serum samples of dextran sulfate sodium (DSS) treated mice and children with IBD. Furthermore, they discovered an increased expression of profibrotic tumor growth factor-b (TGF-β) and platelet-derived growth factor-B (PDGF-B) in colonic epithelial cell culture, along with an increased expression of extracellular matrix (ECM)-related genes and increased motility of colonic fibroblast cells (CCD-18Co) after treatment with recombinant IL-24, suggesting that IL-24 is promoting tissue remodeling [16]. In animal colitis models, IL-20rb knockout (KO) mice were found to have decreased disease activity index and expression of profibrotic TGF-β and PDGF-b compared to WT(wild-type), supporting the same theory [9, 16]. However, this can be misleading, as IL20rb deficiency leads to the loss of not only IL-24 but also IL-19 and IL-20 signaling, as these cytokines share the same receptor [9, 15]. Indeed, results from a recent study using RNA sequencing and Western Blot analysis showed that mice lacking IL-20R expression were more susceptible to DSS-induced colitis, and IL-20 levels were induced during remission in IBD patients and especially in those who responded to anti-TNF treatment [17].In our study, the IL-24 expression was increased in UC patients after treatment with biologics and was statistically related with the Mayo Score (p<0.05). To our knowledge, this is the first study using IHC that suggests a direct relation of IL-24 with the activity of UC, reflecting better outcomes post-treatment and a most likely immunosuppressive role of this cytokine.

IL-19 was proven to interact between the innate immune system and the tissues, promoting angiogenesis and keratinocyte activation [18]. Previous studies support a rather suppressive effect on colonic inflammation through various pathways [10]. Azuma et al., in their animal study, showed that IL-19 deficient mice are prone to developing severe colitis post-treatment. An increased concentration of proinflammatory cytokines (IFN-γ, IL-1β, IL-6, IL-12, and TNF-α) was also found on the colonic tissues [19]. Steinert et al. also demonstrated that DSS colitis was attenuated in IL-19-deficient mice [20]. They have also suggested that a breach of the mucosal barrier by microbes or bacteria-derived products, might be inducing the IL-19 production [20]. In a TNBS (2,4,6-trinitrobenzene sulphonic acid)- induced experimental colitis model in mice with genetic ablation of IL-19, Matsuo et al. found that colitis was exacerbated, with increased presence of interferon-gamma, IL-12 (p40), IL-17, IL-22, and IL-33, and decreased production of IL-4 [21]. Chen et al. showed that murine IL-19 gene therapy, delivered by an attenuated Salmonella strain, alleviates DSS-induced colitis by induction of IL-10 [3]. Older studies have shown that IL-19 activates the transcription of IL-10 [22]. There is only one recent study by Li et al. with controversial results that showed IL-19 overexpression causing aggravation of DSS-induced colitis in IL-19 KO mice [4]. This was explained by the non-specific IL-19 blockade that might be affected by the expression of other ILs that share the same receptor, like IL-24 and IL-20 [4]. Our results showed an increased IL-19 expression in IBD and especially CD patients versus healthy controls, which agrees with previous outcomes from the experimental colitis models (p<0.05).

Only a few human studies investigated the expression of IL-19 in the colonic mucosa. Fonseca Camarillo et al., on a group of 77 Mexican Mestizo patients with IBD, found that IL-19 mRNA was increased versus healthy controls, especially in active CD and less in UC. However, no difference was found between treatment groups [7].

Our results showed an increased IL-19 expression in IBD, especially in the CD group of patients versus healthy controls (p<0.05 and p<0.01, respectively). There was a statistically significant relationship between the pre-treatment IL-19 expression and score reduction in the CD group (p<0.01), suggesting that IL-19 is highly present in active disease and is related to disease progression. More specifically, in the CD group, our study showed a significant difference between IL-19 expression before and after biologic treatment (p<0.05). These results agree with the Fonseca-Camarillo et al. study and support previous findings that IL-19 is increased in active colonic inflammation in CD and its levels are affected by the disease severity [7].

On the contrary, in the UC group, we haven't found any difference in IL-19 expression versus healthy controls. This does not agree with previous studies available [7, 8, 11]. Yamamoto et al. showed that certain polymorphisms in the IL-19 gene could have a protective effect against UC in Mexican patients [11]. However, this was a genetic study aiming to study the role of IL-19 polymorphisms. Fonseca-Camarillo et al. showed increased IL-10-, IL-20-, and IL-20R2-producing cells in active UC patients [8]. We already know that IL-10 is induced by a variety of cytokines, including IL-19, IL-20, and IL-24 [10]. In their cross-sectional study, there was a significant increase in the IL-19 m-RNA levels in active UC vs healthy controls (p<0.05) but no significance between active/inactive UC. What is more, on the protein level, after using immunohistochemistry (IHC), there was no significant difference in expression between active UC and controls. IHC results showed weak staining as well for IL-19 [7]. As results can be misleading, further studies are needed to clarify the expression of IL-19 expression in UC patients in both mRNA and protein level.

A reverse relation was found between ΔIL-19 expression and activity index score reduction in the anti-TNFα and VDZ-treated subgroups of CD patients (p<0.05). A similar relation was found in the whole subgroup of IBD patients (N=59) treated with anti-TNFa agents (p<0.05). This potentially suggests that IL-19 remains high in patients with high activity index scores on their initial presentation that eventually respond to treatment. Equally, we can suggest that IL-19, despite being upregulated in active disease, might be ineffective in refractory cases to medical treatment. Further research is needed to describe the exact actions of this interleukin in CD.

We acknowledge the limitations of this study, which were the relatively small size of the VDZ and USK-treated subgroups. Funding limitations and its' limited use in clinical practice at the time the data/biopsies were collected were the main cause. Many patients that did not attend their follow-up appointments were excluded from the study due to the lack of data regarding their progress.

## Conclusions

Although IL-19 and IL-24 have been studied extensively in other autoimmune diseases, their exact role in IBD is not entirely clear. There are a few animal studies supporting a rather immunosuppressive role of those cytokines; however, there is a lack of adequate human studies to support this. This is one of the first human studies suggesting an immunosuppressive role of IL-24 in UC patients and the first to suggest a direct relation between IL-24 and Mayo Score post-biologic treatment. Additionally, it is one of the first studies that confirms the involvement of IL-19 in IBD-related inflammation, especially in Crohn's disease. A reverse correlation was found between IL-19 expression and HBI score reduction, suggesting that it remains high in refractory cases and can be potentially used as a marker of response to treatment in CD. In addition to this, their colonic expression can potentially assist the histological diagnosis, especially in selected cases with indeterminate findings. Given the limitations of this study, further research is required to fully determine their role in intestinal inflammation. Future studies should focus on prospective follow-up of IL-19 and IL-24 colonic expression of IBD patients for longer surveillance intervals in relation to their serum levels and disease activity.

## Appendices

**Table 1:** Appendix 1 - duration of disease in IBD patients UC - ulcerative colitis; CD - Crohn's disease; IBD - inflammatory bowel disease

Disease	Duration	Frequency	Percent
UC	<3 years	11	22.4
	≥3 years	38	77.6
	Total	49	100.0
CD	<3 years	8	11.1
	≥3 years	64	88.9
	Total	72	100.0

**Table 2:** Appendix 2 - Montreal Classification of IBD patients UC - ulcerative colitis; CD - Crohn's disease; IBD - inflammatory bowel disease

Disease	Classification	Frequency	Percent
UC	S1	16	32.7
	S2	15	30.6
	S3	18	36.7
	Total	49	100.0
	E1	5	10.2
	E2	24	49.0
	E3	20	40.8
	Total	49	100.0
CD	A1	1	1.4
	A2	33	45.8
	A3	38	52.8
	Total	72	100.0
	L1	26	36.1
	L2	14	19.4
	L3	30	41.7
	L3+L4	2	2.8
	Total	72	100.0
	B1	47	65.3
	B2	19	26.4
	B3	6	8.3
	Total	72	100.0

**Table 3:** Appendix 3 - Treatments of IBD patients UC - ulcerative colitis; CD - Crohn's disease; ADA - adalimumab; IFX - infliximab; USK - ustekinumab; VDZ - vedolizumab; IBD - inflammatory bowel disease

Type of disease	Type of treament	Frequency	Percentage	Valid percent
UC	ADA	1	2.0	5.3
	IFX	14	28.6	73.7
	USK	1	2.0	5.3
	VDZ	3	6.1	15.8
	Total	19	38.8	100.0
	Other	30	61.2	
	Total	49	100.0	
CD	ADA	11	15.3	21.2
	IFX	33	45.8	63.5
	USK	6	8.3	11.5
	VDZ	2	2.8	3.8
	Total	52	72.2	100.0
	Other	20	27.8	
	Total	72	100.0	

**Table 4:** Appendix 4 - Pre-treatment IL-19 and IL-24 expression in IBD patients and controls IBD - inflammatory bowel disease; IL-19 - interleukin-19; IL-24 - interleukin-24

Pre-treatment	IL-19			IL-24	
Patient type	Stain intensity	Frequency	Percent	Frequency	Percent
IBD	0	59	48.8	30	24.8
	+	57	47.1	70	57.9
	++	4	3.3	18	14.9
	+++	1	0.8	3	2.5
Total	-	121	100.0	121	100.0
Controls	IL-19			IL-24	
	0	18	90.0	12	60.0
	+	2	10.0	8	40.0
	++	0	0.0	0	0.0
	+++	0	0.0	0	0.0
Total	-	20	100	20	100

**Table 5:** Appendix 5 - Post-treatment IL-19 and IL-24 expression in IBD patients IBD - inflammatory bowel disease; IL-19 - interleukin-19; IL-24 - interleukin-24; anti-TNFa - anti-tumor necrosis factor alpha; USK - ustekinumab; VDZ - vedolizumab

Post-treatment	IL-19				IL-24	
Type of treatment	Type of biologic	Stain intensity	Frequency	Percent	Frequency	Percent
Biologics	Anti-TNFa	0	46	78	29	49.2
		+	13	22	23	39
		++	0	0.0	7	11.9
		+++	0	0.0	0	0.0
	Total-anti-TNFa		59	100.0	59	100.0
	USK	0	6	85.71	2	28.6
		+	1	14.29	4	57.1
		++	0	0.0	1	14.3
		+++	0	0.0	0	0.0
	Total-USK		7	9.9	7	100.0
	VDZ	0	2	40.0	3	60.0
		+	3	60.0	2	40.0
		++	0	0.0	0	0.0
		+++	0	0.0	0	0.0
	Total-VDZ		5	7	5	100.0
	Total biologics		71	-	71	-
Non-biologic treatment		0	35	70.0	25	50.0
		+	15	30.0	23	46.0
		++	0	0.0	2	4.0
		+++	0	0.0	0	0.0
	Total non-biologics		50	100.0	50	100.0

## References

[1] Yadav PK, Chen C, Liu Z (2011). Potential role of NK cells in the pathogenesis of inflammatory bowel disease. J Biomed Biotechnol.

[2] Zhong Y, Zhang X, Chong W (2022). Interleukin-24 Immunobiology and its roles in inflammatory diseases. Int J Mol Sci.

[3] Chen SY, Chu CT, Yang ML (2023). Amelioration of murine colitis by attenuated Salmonella choleraesuis encoding interleukin-19. Microorganisms.

[4] Li Q, Meng F, Ma X (2023). The colonic interleukin-19 aggravates the dextran sodium sulfate/stress-induced comorbidities due to colitis and anxiety. Front Immunol.

[5] Andoh A, Shioya M, Nishida A, Bamba S, Tsujikawa T, Kim-Mitsuyama S, Fujiyama Y (2009). Expression of IL-24, an activator of the JAK1/STAT3/SOCS3 cascade, is enhanced in inflammatory bowel disease. J Immunol.

[6] Norouzinia M, Chaleshi V, Alizadeh AH, Zali MR (2017). Biomarkers in inflammatory bowel diseases: insight into diagnosis, prognosis and treatment. Gastroenterol Hepatol Bed Bench.

[7] Fonseca-Camarillo G, Furuzawa-Carballeda J, Granados J, Yamamoto-Furusho JK (2014). Expression of interleukin (IL)-19 and IL-24 in inflammatory bowel disease patients: a cross-sectional study. Clin Exp Immunol.

[8] Fonseca-Camarillo G, Yamamoto-Furusho JK (2015). Immunoregulatory pathways involved in inflammatory bowel disease. Inflamm Bowel Dis.

[9] Toskas A, Milias S, Papamitsou T, Meditskou S, Kamperidis N, Sioga A (2024). The role of IL-19, IL-24, IL-21 and IL-33 in intestinal mucosa of inflammatory bowel disease: a narrative review. Arab J Gastroenterol.

[10] Chen J, Caspi RR, Chong WP (2018). IL-20 receptor cytokines in autoimmune diseases. J Leukoc Biol.

[11] Yamamoto-Furusho JK, Álvarez-León E, Fragoso JM, Gozalishvilli A, Vallejo M, Vargas-Alarcón G (2011). Protective role of interleukin-19 gene polymorphisms in patients with ulcerative colitis. Hum Immunol.

[12] Yamamoto-Furusho JK, Fonseca-Camarillo G (2015). Genetic markers associated with clinical outcomes in patients with inflammatory bowel disease. Inflamm Bowel Dis.

[13] Bevivino G, Monteleone G (2018). Advances in understanding the role of cytokines in inflammatory bowel disease. Expert Rev Gastroenterol Hepatol.

[14] Toskas A, Milias S, Delis G, Meditskou S, Sioga A, Papamitsou T (2023). Expression of IL-21 and IL-33 in intestinal mucosa of inflammatory bowel disease: an immunohistochemical study. Diagnostics (Basel).

[15] Fonseca-Camarillo G, Furuzawa-Carballeda J, Llorente L, Yamamoto-Furusho JK (2013). IL-10-- and IL-20--expressing epithelial and inflammatory cells are increased in patients with ulcerative colitis. J Clin Immunol.

[16] Ónody A, Veres-Székely A, Pap D (2021). Interleukin-24 regulates mucosal remodeling in inflammatory bowel diseases. J Transl Med.

[17] Chiriac MT, Hracsko Z, Günther C (2024). IL-20 controls resolution of experimental colitis by regulating epithelial IFN/STAT2 signalling. Gut.

[18] Kako F, Gabunia K, Ray M (2016). Interleukin-19 induces angiogenesis in the absence of hypoxia by direct and indirect immune mechanisms. Am J Physiol Cell Physiol.

[19] Azuma YT, Matsuo Y, Kuwamura M (2010). Interleukin-19 protects mice from innate-mediated colonic inflammation. Inflamm Bowel Dis.

[20] Steinert A, Linas I, Kaya B (2017). The stimulation of macrophages with TLR ligands supports increased IL-19 expression in inflammatory bowel disease patients and in colitis models. J Immunol.

[21] Jordan WJ, Eskdale J, Boniotto M, Lennon GP, Peat J, Campbell JD, Gallagher G (2005). Human IL-19 regulates immunity through auto-induction of IL-19 and production of IL-10. Eur J Immunol.

[22] Matsuo Y, Azuma YT, Kuwamura M (2015). Interleukin 19 reduces inflammation in chemically induced experimental colitis. Int Immunopharmacol.

